# Historical Text Line Segmentation Using Deep Learning Algorithms: Mask-RCNN against U-Net Networks

**DOI:** 10.3390/jimaging10030065

**Published:** 2024-03-05

**Authors:** Florian Côme Fizaine, Patrick Bard, Michel Paindavoine, Cécile Robin, Edouard Bouyé, Raphaël Lefèvre, Annie Vinter

**Affiliations:** 1 LEAD-CNRS, Université de Bourgogne, 21000 Dijon, France; patrick.bard@cnrs.fr (P.B.); annie.vinter@u-bourgogne.fr (A.V.); 2 Archives Départementales de Côte d’Or, 21000 Dijon, France; cecile.robin@inp.fr (C.R.); edouard.bouye@cotedor.fr (E.B.); 3 Institut National du Patrimoine, 75002 Paris, France; 4 Société Nationale des Chemins de fer Français, 93200 Saint Denis, France; raffael.lefevre@gmail.com

**Keywords:** deep learning, line segmentation, instance segmentation, Mask-RCNN, U-Net, historical document analysis

## Abstract

Text line segmentation is a necessary preliminary step before most text transcription algorithms are applied. The leading deep learning networks used in this context (ARU-Net, dhSegment, and Doc-UFCN) are based on the U-Net architecture. They are efficient, but fall under the same concept, requiring a post-processing step to perform instance (e.g., text line) segmentation. In the present work, we test the advantages of Mask-RCNN, which is designed to perform instance segmentation directly. This work is the first to directly compare Mask-RCNN- and U-Net-based networks on text segmentation of historical documents, showing the superiority of the former over the latter. Three studies were conducted, one comparing these networks on different historical databases, another comparing Mask-RCNN with Doc-UFCN on a private historical database, and a third comparing the handwritten text recognition (HTR) performance of the tested networks. The results showed that Mask-RCNN outperformed ARU-Net, dhSegment, and Doc-UFCN using relevant line segmentation metrics, that performance evaluation should not focus on the raw masks generated by the networks, that a light mask processing is an efficient and simple solution to improve evaluation, and that Mask-RCNN leads to better HTR performance.

## 1. Introduction

Archives around the world contain many manuscript documents that have been preserved over the centuries. These documents are an invaluable source of information for historians, genealogists, etc., but also for anyone who wants to know more about specific points in the past. In recent years, a major digitization project has been undertaken by the French National Archives [1] to make these documents accessible to as many people as possible. They are now available online in the form of image files but as the writing and the language have largely evolved over the centuries, these historical texts remain, for a large part of them, difficult to read for a noninitiated person. The intervention of a paleographer is then necessary to transcribe them, which is costly and time-consuming. Removing these barriers to access is what motivates most of the scientific work devoted to automating the transcription of such documents.

**Figure 1:**
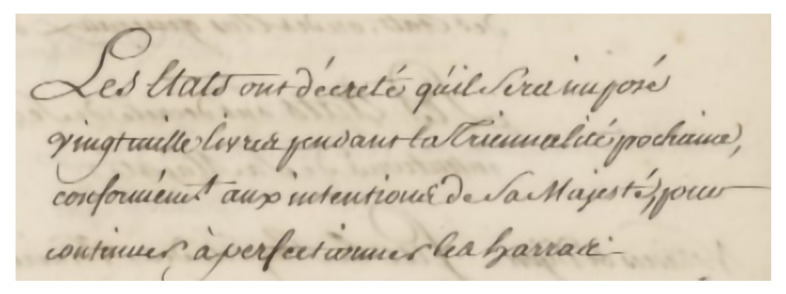
Example of historical text to be transcribed (drawn from the Deliberation Registers of the States of Burgundy) courtesy of the Archives of the Côte d’Or Departmental Council, Copyright 2023 (©/CD21/F.PETOT/2023).

For the sake of illustration, Figure 1 reproduces some handwritten lines extracted from a historical text (Deliberation Registers of the States of Burgundy, Dijon, France). This example makes it clear that reading these few lines is not that easy. The difficulty may lie in the topokinetic component [2], i.e., the overall spatial organization of the handwriting. Some letters may occupy more than one line, the spacing between the lines may not be regular and even, and some lines may cross each other. Because the shapes of the letters themselves have changed greatly over time and are subject to individual differences, reading difficulties may also involve the morphokinetic component of handwriting, i.e., the shape of the letter itself [2]. The challenge of automatically transcribing ancient documents therefore remains a largely unsolved problem.

Different approaches have been used to solve this problem [3]. Among them, a common and competitive approach distinguishes two phases in the process [4]. The first phase aims at the segmentation of the lines, and the second at the transcription of the text itself. From the digital image files, the segmentation phase consists of extracting the handwritten lines with the most accurate clipping possible. These subimages are then used as input for the transcription process, which deciphers the letters/words. The present paper focuses on the first phase, the segmentation phase, applied to historical documents.

Generally speaking, the segmentation of text lines has been studied for many years and many techniques have been proposed, initially with mathematical approaches without learning [3]. Although they are still being improved using an energy minimization procedure ([5,6]), they can hardly compete in terms of performance achieved with the artificial intelligence (AI)-based approaches that will be used in the present work.

With artificial neural network learning, it is now possible to transcribe large volumes of documents automatically, quickly, and inexpensively. For example, this approach led to the creation in 1998 of the MNIST (Mixed National Institute of Standards and Technology) database [7]. This database of 70,000 handwritten digits has been the basis for the development of automatic sorting systems for postal codes and checks. It is now possible to automatically transcribe entire pages of printed or handwritten text, as long as they are presented in standard formats with good legibility. However, these techniques reach their limits as soon as the form of the text becomes more complex, such as in historical texts (Figure 1).

Recent algorithms developed within the framework of the deep learning approach have revitalized this area of research (see [8] for a complete review). Deep learning algorithms have led to significant improvements in the processing of complex images and especially in object detection, such as the Yolo [9], SSD [10] or Fast-RCNN [11] algorithms. For example, [12] used the YoloV5 algorithm to demonstrate its ability to distinguish and identify the main body of a text, the text in the margins, and the common headings. Each region of interest in the text is delimited by bounding boxes. However, while these object detection networks are very relevant for this type of application, they are much less suitable for accurate line segmentation because the bounding boxes generated are defined by very simple polygons of typically four points.

Other deep learning algorithms have been developed to perform semantic segmentation and instance segmentation. Semantic segmentation aims to distinguish classes of objects from each other, pixel by pixel, while instance segmentation separates different classes of objects, *and* also individual objects within the same class. Thus, instance segmentation requires one output by object, not just by class. The main architectures used for this purpose, originally developed for applications in the medical domain, are the U-Net [13] and the Mask-RCNN [14] networks, which are based on different philosophies, as we will see in the next section. The improvements they have achieved over classical AI algorithms in domains that demand high accuracy and speed, such as medicine and automatic driving, are opening up new possibilities for other domains, in particular the automatic transcription of old handwritten documents.

In this article, we confront these two families of neural network models for line segmentation of historical documents in the context of text transcription. The first family (U-Net networks) provides semantic segmentation as output and requires post-processing to generate instance segmentation. The second family (represented by Mask-RCNN in our study) includes networks optimized to perform instance segmentation directly. Our guiding hypothesis is that an instance segmentation network would outperform semantic segmentation networks for text line segmentation of historical documents, leading to better handwritten text recognition (HTR) performance. Among the instance segmentation networks, Sharma et al. [15] distinguishes between those that are “proposal-based”, using the bounding box technique before segmentation, and those that are “proposal-free”, using the clustering or grouping technique at the pixel level to generate object masks. Mask-RCNN belongs to the first category. We present an original way of exploiting the Mask-RCNN architecture, which differs from the main work we currently know that uses this network to extract lines of text in historical documents [16]. We will first describe the U-Net and Mask-RCNN networks and introduce the work of [16], which allows us to give the rationale for our choice to select the study of [17] as the source of our performance comparisons. U-Net and Mask-RCNN networks are then compared in the context of text line segmentation for text transcription using different public databases (cBaD 2017 READ-Complex [4], DIVA-HisDB [18], HOME-Alcar [19]) and a private database (the Deliberation Registers of the States of Burgundy, hereafter DRoSB). In addition, we propose an alternative to the current way of evaluating network segmentation performance, while retaining the existing measures. Finally, the respective performance of both families of segmentation networks in terms of HTR will also be analyzed.

## 2. Related Work

This section introduces and compares U-Net and Mask-RCNN on a more conceptual level as representatives of semantic versus instance segmentation networks. Then, recent studies exploring the potential of Mask-RCNN for extracting lines of text in historical documents are presented.

### 2.1. U-Net

Three state-of-the-art networks based on the U-Net architecture [13], namely, dhSegment [20], ARU-Net [21] and Doc-UFCN [22], are compared in [17], showing the efficiency of U-Net-based architectures, their current dominance for text line segmentation, and their limitations.

**Figure 2:**
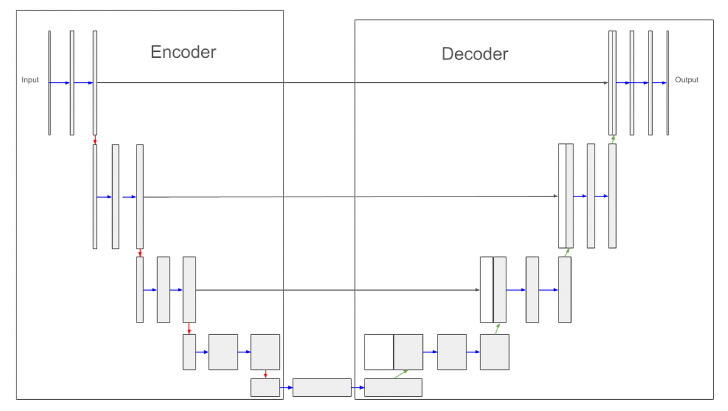
U-Net architecture, → CNN Block, ↓ downsampling, ↑ upsampling, → skip connection (adapted from [13]).

U-Net is a fully convolutional neural network (FCNN) [23] with a U-shape, as shown in Figure 2. It consists of two main parts, an encoder and a decoder. The encoder is a kind of contraction path. Composed of successive CNN blocks and downsampling blocks between the CNN blocks, it reduces the spatial resolution of the image in order to obtain a more abstract representation. Conversely, the decoder is an expanding path. It also consists of CNN blocks, but with upsampling blocks between the CNN blocks. It expands the spatial resolution of the abstract representation to match the target representations. In the case of text line segmentation, the target representations can be baselines or masks that, when applied to the original image, extract the desired text lines.

Blocks with the same spatial dimension in the encoder and decoder are connected by skip links that preserve the features obtained at different resolutions. These specifications require nonoverlapping masks as ground truth to train the network. In the inference phase, a post-processing step is required to separate the overlapping masks or to recover the text line boundaries if the output is the baseline [20]. For example, considering the dhSegment neural network, as indicated by [20], post-processing corresponds to the following four steps: thresholding, morphological operations, connected component analysis, and shape vectorization. Such processes are not necessary with Mask-RCNN.

### 2.2. Mask-RCNN

**Figure 3:**
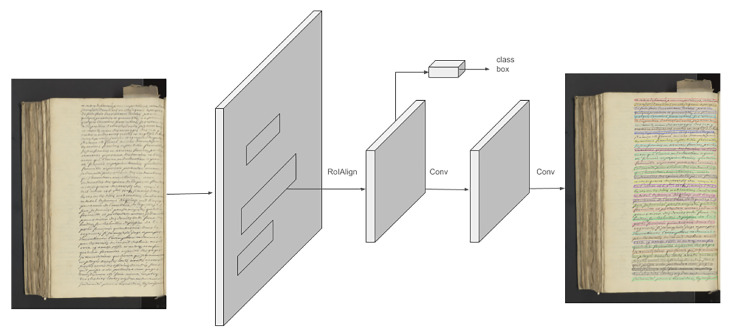
Mask-RCNN architecture. On the left, the color image is the input of the network and on the right, and the segmented image is the output (adapted from [14]).

Mask-RCNN [14] is built on top of Faster-RCNN [24], an object detection network. Its principle is illustrated in Figure 3.

Faster-RCNN is an object detection network itself, consisting of an FCNN backbone used as a region proposal network and the detection stage of Fast-RCNN. It proposes regions of multiple scales and aspect ratios. Each region has a bounding box coordinate output consisting of four values, x,y position, height and width of the bounding box, and another output with the probability that a given region contains an object. These regions are called anchors. The original paper [14] proposed three scales and three aspect ratios per location, i.e., nine anchors per region. The region proposal step provides a set of regions containing objects, and then the classification step performs a classification to determine which objects are present in each proposed region. Thus, Faster-RCNN provides two outputs, a class label and a bounding box offset. Mask-RCNN adds a third output, a binary mask for each region of interest (RoI). The loss is calculated for each RoI and is composed of a classification loss, a bounding box loss, and a mask loss. With this method, there is no competition between classes in each bounding box. In addition, a sigmoid activation is used so that the mask loss is a binary loss. Mask-RCNN also adds an RoI alignment signal, which is used to avoid misalignment between the RoI and the extracted features.

### 2.3. Conceptual Comparison between U-Net and Mask-RCNN

Regarding line segmentation in the context of HTR, the most important thing is to separate the instances of a single class (text line). Since semantic segmentation tends to agglomerate very spatially close objects of the same class, we claim that it is preferable to use an instance-based segmentation model in the case of historical documents because of the high occurrence of overlapping lines and heterogeneity in the shape and size of the ascending and descending strokes in these documents.

As mentioned earlier, U-Net performs semantic segmentation (separation of different classes), and can also perform instance segmentation (separation of instances within the same class) by adding different post-processing stages. Mask-RCNN performs instance segmentation directly, and therefore does not require an additional post-processing step to separate objects of the same class. In addition, Mask-RCNN can easily handle overlapping masks because it has as many outputs as there are detected objects, and each output is independent. This is especially useful for performing text line segmentation in historical documents, where there are many overlapping text lines. For each mask predicted by Mask-RCNN, the only thing to do to isolate the lines is to apply a logical “and” between the image and the mask.

Vuola et al. [25] compared U-Net and Mask-RCNN networks in the medical field for the segmentation of general nuclei instances in microscopy images. As expected, while U-Net provides more accurate semantic segmentation, Mask-RCNN is better at object separation. The superiority of Mask-RCNN for object separation was recently confirmed in two other applications [26,27]. In our context, the segmentation of text lines is performed for the purpose of text recognition, so the priority is to obtain well-separated lines. The accuracy of the masks is less critical than in the medical domain. However, object separation is of real importance.

### 2.4. Mask-RCNN and Text Line Extraction in Historical Documents

To our knowledge, the first attempt to apply Mask-RCNN to text line segmentation in historical documents was made by Zhao et al. [28] with ancient Tibetan documents. The authors used two versions of this network in their comparative experiments to validate their improvement of their SOLOv2 network [29] on these challenging documents. The poor segmentation performance reported for the Mask-RCNN approach in this study would discourage pursuit in this direction. However, we can point out that the line-level layout of the Tibetan historical texts, which, in particular, allows large spaces between words of the same line both horizontally and vertically, lends itself well to the pixel-level clustering or grouping technique for object mask generation (as used in SOLOv2), but not to the bounding box technique (as used in Mask-RCNN).

In fact, the other study we know of, where Mask-RCNN was used for text line segmentation in historical documents with Latin-based or Arabic scripts [16], gives much more encouraging results. In this study, the network was trained to segment fragments of text lines into overlapping patches, which are then merged together to reconstruct the entire page with its multiple segmented text lines. The authors decided to compare the performance of Mask-RCNN with that of different segmentation methods reported in Task-3 of the ICDAR 2017 competition, using two databases of medieval documents, DIVA-HisDB and ICDAR 2015-HTR. Their results showed that a purposely designed Mask-RCNN outperformed the best-performing methods presented in ICDAR 2017. The study also demonstrates that Mask-RCNN can successfully segment texts from a challenging database, such as the VML-AHTE database made of Arabic manuscripts with numerous diacritics.

These encouraging results confirm our preliminary data showing that Mask-RCNN is a promising network for text line segmentation of historical documents, at least with Western scripts [30,31]. In parallel with [16], we actually developed another approach to exploit the Mask-RCNN architecture for the same purpose, while overcoming some of the weaknesses of their own approach. Indeed, the networks with which these authors challenged Mask-RCNN are not recent ones and, unlike U-Net networks, cannot be considered as state-of-the-art artificial neural networks. In addition, at least one of the databases they choose for the comparative evaluation (ICDAR 2015-HTR) is not sufficiently challenging, since it excludes all the annotations contained in the margins of the text, which yet contribute to the complexity of the segmentation. These limitations justify our choice to use databases and U-Net networks similar to those of Boillet et al. [17] to evaluate our Mask-RCNN network. This way, we anchor our work in the state of the art better than [16] did. In addition, our implementation of the Mask-RCNN network differs in two important ways from [16]. These authors performed a binarization of the images and separated the segmented data into patches before merging them. These operations are likely to introduce biases. To avoid these biases, in our work, we replaced binarization with thresholding to help the model distinguish the lines on the page from those visible in the transparency on the back of the page, and we did not use patches (no post-processing). We favored the direct processing of whole pages without post-processing during training and inference, which is much simpler than other approaches and also has the advantage of reducing computational complexity.

## 3. Materials and Methods

### 3.1. Data Preparation for the Ground Truth of the Deliberation Registers of the States of Burgundy

Our Mask-RCNN neural network was trained with the DRoSB dataset. These are French documents dating from the XVIIIth century that record the decisions of the States of Burgundy, which, under the Old Regime, governed the province together with the royal power. These texts consist of 120 double pages of 20 to 60 lines each. They cover several decades with a fairly homogeneous layout without being identical on each page. The pages can contain a title, the body text and margin notes. The lines have a variable spacing (generally quite wide, but some pages are denser). There are words between the lines, which we decided to treat as a separate line. Transparency (text on the back of the page that you can see) is very variable; some pages do not have any and others are hard to read because of this transparency. Above all, the challenging recurrent feature of these texts is the presence of long ascending and descending strokes, provoking numerous overlapping lines. Stains on the pages are rare, but they do exist. Some of the documents are available on https://iiif.lettres-en-lumieres.org/ (accessed on 1 September 2023).

Mask-RCNN, like any other segmentation neural network, needs for its training characteristic images to be associated with segmentation masks that cut out as precisely as possible the objects to be segmented (in this case, the text lines). The goal is to get as close as possible to the outline of the handwritten characters on a line, which have heterogeneous shapes and sizes. Given this variability, we sustain that polygons provide the best representation of the outline of a line.

#### 3.1.1. Creation of Segmentation Masks

**Figure 4:**
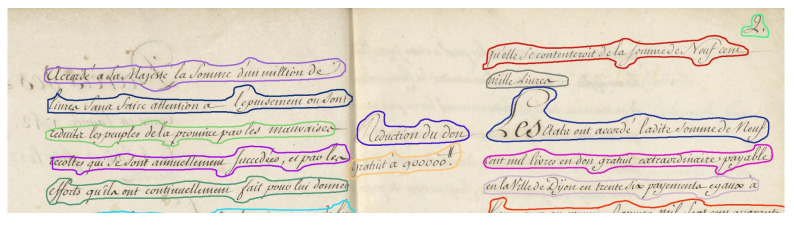
Example of annotations in “polygon” mode of a few lines extracted from the DRoSB dataset, courtesy of the Archives of the Côte d’Or Departmental Council, Copyright 2023 (©/CD21/F.PETOT/2023).

Each image was manually annotated with a large mask, with the only constraint that the lines had to be well separated, without including parasite data. Figure 4 shows an example of annotation in “polygon” mode of a text drawn over an image from the DRoSB dataset. As can be seen, the masks fit closely to the lines of text, following the contours of the ascending and descending handwritten strokes.

For overlapping lines, we took all the pixels from the text line of interest and as few pixels as possible from the other lines. Note that overlapping masks were permitted.

#### 3.1.2. Image Format for Training and Inference

As preprocessing of the images used for training, we performed thresholding by calculating the binary image using Otsu binarization [32], then multiplying this image by the original image. With this method, we retained all the information contained in text line, but removed the background. This thresholding step was not necessary, but it did slightly increase performance. Finally, we fed the Mask-RCNN network with these thresholded images on a scale of 256 gray levels as inputs.

For training purposes, all images were resized to a specific definition without preserving the aspect ratio. We trained the network with mages of 1024 or 2048 pixels on both width and height, respectively. The tests revealed that a dimension of 1024 × 1024 pixels on a double page gave poor results in case of isolated words between lines. Since the DRoSB dataset contains many isolated words between the lines, overlapping the top or bottom lines, this particular problem was relevant in our case. We therefore chose a dimension of 2048 × 2048 pixels. This resizing did not respect the width-to-height ratio and led to a distortion of the image, but in our case, it was not detrimental. The recognized masks were then resized to the size of the original image, which made it possible to extract the text lines to be transcribed with very good precision. We set the maximum number of instances (lines) displayed in each image to 600 in order to be able to handle very dense pages or tables without wasting memory. In the cBaD READ-Complex database (see next section), there is an average of 80 lines and a maximum of 472 lines per page, which means that a limit of 600 lines allows it to handle all pages of cBaD, but also pages that may be denser than those present in cBaD.

### 3.2. Choice of Public Databases and U-Net Networks

**Figure 5:**
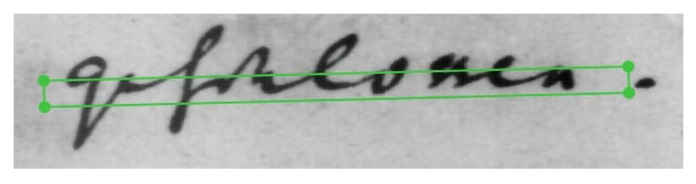
Example of poor mask precision in cBaD 2017 annotation, public database. The green box represents the ground truth’s mask.

To complete the DRoSB database and to compare the performance of Mask-RCNN with U-Net networks, we added the cBaD 2017 database provided for the ICDAR 2017 conference competition [4], the DIVA-HisDB, and the HOME-Alcar database for the training of Mask-RCNN. These databases are diversified, draw on a large number of historical texts, and allow us to have a wide variety of annotations. Boillet et al. [17] performed an in-depth comparative study of the use of U-Net on a large number of databases. Their results made it clear that the cBaD database of ICDAR 2017 is the most discriminative for the segmentation of historical texts, especially the READ-Complex subset. This justifies our choice of this dataset, although it contains very poor segmentation data, as can be seen in Figure 5. In fact, the lines are segmented with rather imprecise bounding boxes, with cutting letters going up and down. Even if it is sufficient to detect the baseline, it remains unsuitable for extracting lines of text for transcription. Therefore, we also chose DIVA-HisDB, which provides more complex polygons, very close to the line, but not the closest. For this reason, to match the annotation style of DIVA, we applied our LMP algorithm described in Section 3.3.3 to the Mask-RCNN output masks. With this method, we obtained masks as close as possible to the text lines recognized by Mask-RCNN. We also applied a dilation to the masks (with a kernel of 2 × 8 and 4 iterations) to obtain masks slightly larger than the text lines. Finally, we included the HOME-Alcar database in our study because this dataset is somewhat in between the cBaD database (it contains cBaD style annotations) and what we would expect in terms of masks (with polygons that take in all the information of the line). For this dataset, we evaluated Mask-RCNN on the raw output masks.

On the basis of the Boillet et al.’s results [17], we selected three state-of-the-art U-Net architecture networks: dhSegment [20], ARU-Net [21], and Doc-UFCN [22], to compare the performance of Mask-RCNN. In the present study, we used the performance data reported by these authors in Tables 4 and 5 of their article (note, however, that we replicated these trainings in a pilot study and found approximately the same values, and, most importantly, in the same hierarchy). In our comparisons, we also included the Dilated-FCN network tested by [33] for line segmentation on cBaD and DIVA-HisDB. Unfortunately, these authors only reported a pixel-level metric, the F1-score (see the next section). The comparison of this U-Net network with Mask-RCNN is therefore more limited.

### 3.3. Evaluation of Segmentation Performance of the Tested Networks

To evaluate the performance of Mask-RCNN against the U-Net networks, the selected metrics were at both the pixel and object level, as convincingly introduced in [17].

#### 3.3.1. Pixel-Level Metrics

Pixel-level metrics evaluate the proportion of pixels that are well classified. They provide information about the overall quality of the segmentation, but not about the individual masks. The formulas used are detailed in (1) to (4), where TP (true positive) is the number of well-classified pixels, FP (false positive) is the number of pixels classified as lines when they are actually background, and FN (false negative) is the number of pixels classified as background when they are actually lines.

(1) $$Precision\left[P\right]=\frac{TP}{TP+FP}$$

(2) $$Recall\left[R\right]=\frac{TP}{TP+FN}$$

(3) $$F1-\mathrm{Score}=\frac{2PR}{P+R}$$

(4) $$\mathrm{Intersection over Union}\left[IoU\right]\;=\frac{TP}{TP+FP+FN}$$

In this context, a positive element is a pixel that belongs to a line-matching mask. The *P*, *R*, F1-score, and IoU values are in the range [0, 1] where 1 is the best score. Precision is the rate of correct positive predictions, while recall is the rate of correctly predicted positive elements. Although they are of interest in their own right, precision and recall are often intermediate results in the calculation of more important measures. The F1-score combines them according to their harmonic mean, and measures the ability of the model to predict positive items in terms of precision and recall. We want this score to be as high as possible. It tends to represent the average performance of the network. The original definition of the fourth pixel-metric, intersection over union (IoU), is the ratio of the area of the intersection of the overlapping ground truth mask and the predicted mask to the area of the union of these two masks. Its calculation is summarized in (4). We also want this score to be as high as possible. While the F1-score is a metric that combines the precision and the recall, the IoU score is a metric that highlights the proportion of true positive pixels compared to all pixels present in the mask. The IoU average on a dataset allows the comparison of neural networks for their worst case while the F1-score allows the comparison of their average results. As a result, the difference between the IoU and F1-scores increases when the performance of the neural network is poor.

#### 3.3.2. Object-Level Metrics

Boillet et al. [17] also proposed to compute object-level metrics because they showed that the pixel-level metrics are not sufficient to evaluate text line segmentation networks. In fact, pixel-level metrics only evaluate how well the network extracts the searched pixels, but do not provide any information about whether the separated objects are grouped or not.

The object-level metrics are defined in (5) and (6). The detection threshold is a hyperparameter of Mask-RCNN networks ranging from 0 to 1. The networks generate only those masks whose detection confidence level is greater than this threshold. $P_{k}$ and $R_{k}$ are the precision and the recall with a detection threshold of k, respectively. $TP_{k}$ and $Total_{k}$ are the true positive and total detection with a detection threshold of k, respectively, and $Total_{GT}$ corresponds to the total of ground truth detection.

(5) $$P_{k}=\frac{TP_{k}}{Total_{k}}$$

(6) $$R_{k}=\frac{TP_{k}}{Total_{GT}}$$

**Figure 6:**
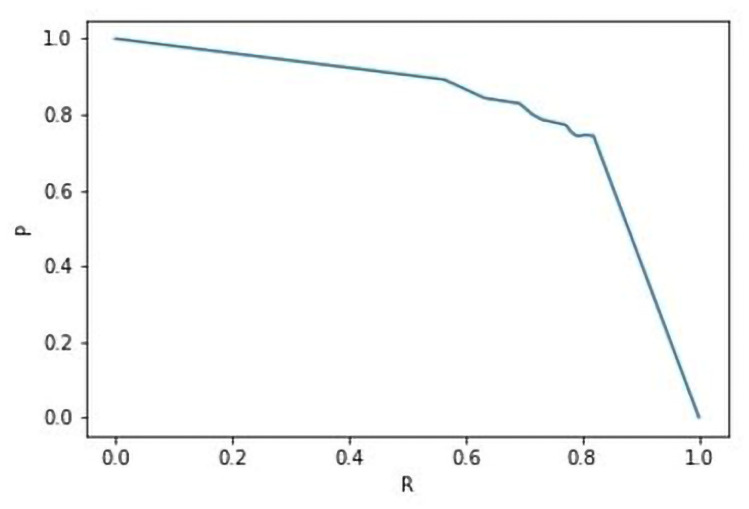
Precision–recall curve.

In addition to these two metrics, we calculated the averaged precision (AP) from the PASCAL VOC Challenge [34] at different IoU thresholds (0.5, 0.75), and the mean of the AP (AP@[0.5,0.95]) from 0.5 to 0.95. The average precision was calculated as the area under the precision–recall curve, as shown in (7), where $P=\{P_{0},..,P_{k},..P_{1}\}$ and $R=\{R_{0},..,R_{k},..R_{1}\}$. This is illustrated in Figure 6. Like the pixel-level metrics, $P_{k}$, $R_{k}$ and AP vary between [0,1], with 1 being the best score.

(7) $$AP=\int_{0}^{1}P\left(R\right)\;dR$$

#### 3.3.3. Light Mask Processing for Evaluation When Using the DRoSB Database

**Figure 7:**
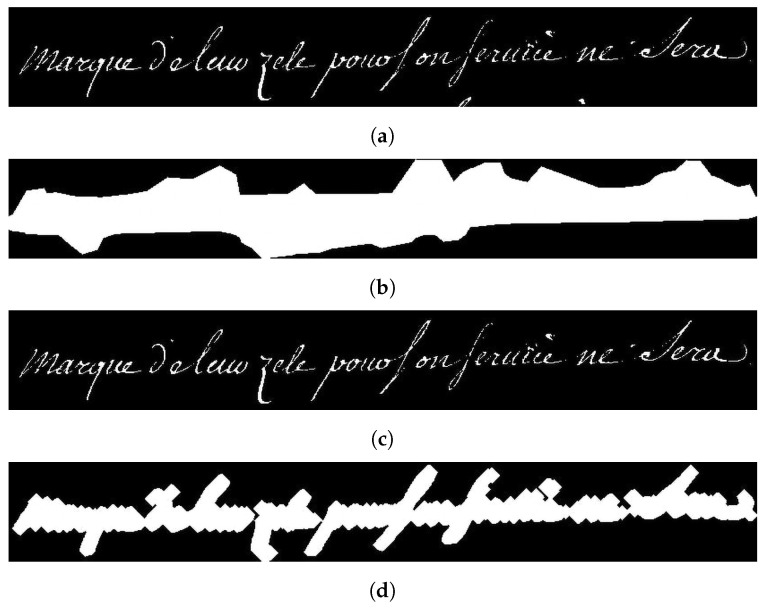
Illustration of the different steps of the Light Mask Processing (LMP) algorithm. (**a**) Binarized images of one line; (**b**) output mask of the network; (**c**) result of multiplication between mask and line; (**d**) mask with minimum size to ensure that all pixels are connected. (Applied to a line of text from the DRoSB, courtesy of the Archives of the Côte d’Or Departmental Council, Copyright 2023 (©/CD21/F.PETOT/2023)).

Ideally, the segmentation masks generated by neural networks should match the ground truth. Depending on the database, ground truths have more or less wide masks. In the case of wide masks, relevant pixels that correspond to the text itself are in the minority compared to the other pixels of the mask that correspond to the background of the document. To avoid this bias, we developed the Light Mask Processing (LMP) algorithm, as illustrated in Figure 7.

In detail, the image is first binarized using the Otsu algorithm (Figure 7a), and then multiplied by the generated mask (Figure 7b). These steps are similar to those described for foreground pixel accuracy (FgPA) in [35]. However, we are not proposing a new metric with the LMP, as [35] did with the FgPA, but a new mask processing in order to make existing metrics more accurate. The result of this multiplication step (Figure 7c) is then subjected to a closure operation derived from morphological mathematics (Figure 7d). This step allows LMP to be used in metrics that focus on object segmentation, such as those detailed in the previous section. Finally, we apply a logical “and” between the processed mask and the generated mask to obtain a constant mask that is larger than the letters, but without any noisy data.

**Figure 8:**
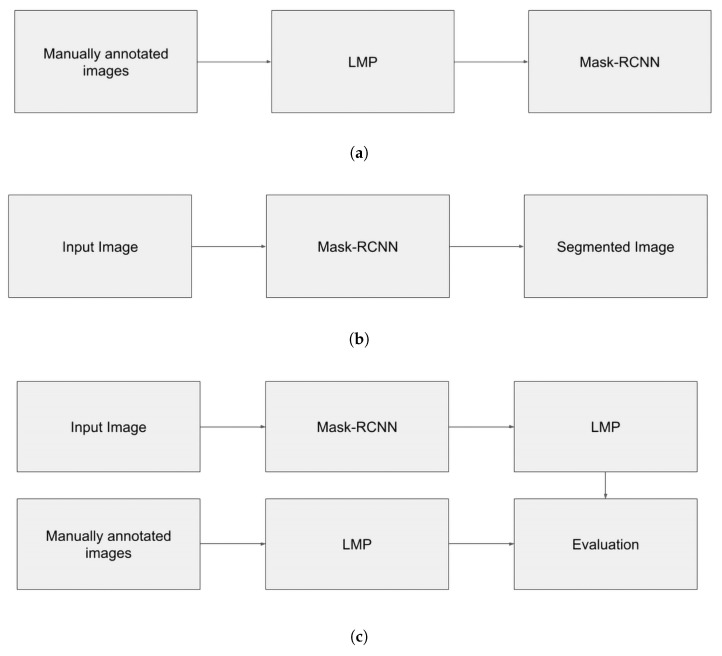
Panels (**a**–**c**) show where the LMP is applied in our processing pipeline. (**a**) Use of LMP during network training; (**b**) Use of Mask-RCNN for inference; (**c**) Use of LMP during validation.

LMP does not add or remove any pixels in comparison to the pixels present in the binarized image multiplied by the raw mask. Although the processing is sensitive to the quality of the binarization, the noise caused by a bad binarization is the same for all models evaluated. In addition, regardless of the quality of the binarization, the LMP produces masks that are closer to the text and thus will achieve a more reliable evaluation than when using raw masks. Finally, to perform the evaluation, in order to use the same algorithm as [17], after the LMP operation, a closing iterative operation with a progressive increase in the kernel size (of 1 pixel) is applied until all the pixels belonging to the mask touch each other. This operation adapts to the shape of the line to be cut. It should be noted that this step was necessary because we decided to use the scripts of [17] in order to apply the same measures as those authors. Figure 8 shows the different use cases of the LMP processing.

LMP is thus applicable to any line segmentation algorithm. It allows the transformation of any network output (either a binarization oriented on the pixels of the line or a mask surrounding the text) into a minimal polygon encompassing the text of the line. It allows the comparison of any type of network under the same conditions, focusing only on the data relevant to line transcription.

### 3.4. Implementation of Networks

We implemented Mask-RCNN using the tensorflow object detection API from the official github (https://github.com/tensorflow/models/blob/master/research/object_detection/configs/tf2/mask_rcnn_inception_resnet_v2_1024x1024_coco17_gpu-8.config (accessed on 15 January 2022)). We performed three studies. First, we compared the line segmentation performance of Mask-RCNN with that reported by [17] in their Tables 4 and 5 for three U-Net networks on different historical databases, and also by [33] for the Dilated-FCN network. Second, we compared the performance of Mask-RCNN and the better performer among these U-Net networks on the DRoSB dataset. For each of these two studies, the evaluation was performed using the two metrics already discussed, the pixel-level metrics and the object-level metrics. Finally, since the ultimate goal of historical document analysis is the handwritten text recognition (HTR), a third study is dedicated to the implementation of the complete processing chain, comparing the transcription performance associated with either the Mask-RCNN segmentation or a U-Net network segmentation. We applied a hyperparameter tuning to the Mask-RCNN network, testing different anchor box scales and ratios. We found that small scales and horizontal ratios gave better performance. Therefore, we chose the following parameter scales: [0.25, 0.5, 1.0], and aspect_ratios: [1.0, 2.0, 4.0, 8.0]. This implementation did not include any post-processing. In Study 1, the implementation of the U-Net networks realized by the authors to whom we refer in terms of segmentation performance included post-processing (see [17,33]). In Study 2, we implemented Doc-UFCN using the default hyperparameters of the models provided by the author (https://gitlab.com/teklia/dla/doc-ufcn (accessed on 1 September 2022)). No hyperparameter tuning process was performed for this network (which includes post-processing), since it is already optimized for text line segmentation.

### 3.5. Computational Requirements

Currently, all trainings are conducted on an NVIDIA A100 80 GB board for a duration of 4 h on average for 10,000 training steps. The inferences are run on a machine equipped with a 1080ti graphics board with 12 GB of VRAM. Note that a transcription model based on a Transformer network is implemented on the same board. Only 4 to 6 GB of VRAM are actually needed for segmentation, depending on the number of lines present in the document. We did not perform any pruning, either for export with TensorRT or for quantization. These techniques can reduce both memory requirements and inference time. Nevertheless, we used half-precision to reduce the memory requirement. Under these conditions, it takes about 1 second to segment a page, which is quite long, but still satisfactory in the context of full-text transcription.

## 4. Results

We performed three studies. First, we compared the line segmentation performance of Mask-RCNN with that reported by [17] in their Tables 4 and 5 for three U-Net networks on different historical databases, and also by [33] for the Dilated-FCN network. Second, we compared the performance of Mask-RCNN and the better performer among these U-Net networks on the DRoSB dataset. For each of these two studies, the evaluation was performed using the two metrics already discussed, the pixel-level metrics and the object-level metrics. Finally, since the ultimate goal of historical document analysis is the handwritten text recognition (HTR), a third study is dedicated to the implementation of the complete processing chain, comparing the transcription performance associated with either the Mask-RCNN segmentation or a U-Net network segmentation.

### 4.1. Study 1: Mask-RCNN against U-Net Networks on cBaD 2017 READ-Complex, DIVA-HisDB and HOME-Alcar

The results of the comparison between ARU-Net, dhSegment, Doc-UFCN, Dilated-FCN, and Mask-RCNN using pixel-level metrics on cBaD 2017 READ-Complex, DIVA-HisDB, and HOME-Alcar are reported in Table 1. Recall that the segmentation performance of the first three U-Net networks are drawn from [17] and that of the Dilated-FCN network from [33].

Whether we consider the IoU measure or the F1 score, the same pattern of results emerges from Table 1 for cBaD READ-Complex: The best performance is shown by ARU-Net (0.73 and 0.81, respectively), followed by Mask-RCNN (0.64 and 0.73, respectively). For DIVA-HisDB, three U-Net-based networks (ARU-Net, Doc-UFCN and Dilated-FCN) performed better than Mask-RCNN. For the HOME-Alcar database, Mask-RCNN outperformed all U-Net networks for the IoU measure, and was close to that of ARU-Net for the F1-score (0.91 and 0.94, respectively). Thus, despite satisfactory performance, Mask-RCNN does not offer an advantage over U-Net architectures for line pixel identification, as the latter perform well at this task globally, for which they are suitable.

In contrast, as expected, using object-level metrics, Table 2 clearly demonstrates the superiority of Mask-RCNN on the three targeted U-Net based networks, regardless of the average precision threshold and the database. With cBaD READ-complex, DIVA-HisDB, and HOME, the best performance was systematically presented by Mask-RCNN at 0.5 (AP@0.5: 0.87, 0.96, and 0.98, respectively), at 0.75 (AP@0.75: 0.34, 0.60, and 0.86, respectively) and for the mean of the average precision between 0.5 and 0.95 (AP@[0.5,0.95]: 0.42, 0.55, and 0.65, respectively). These results highlight the importance of evaluating networks on both types of metrics. The clear advantage of Mask-RCNN becomes apparent only in the accurate separation of text lines, which is the most important major prerequisite for high-performance HTR. Among the U-Net networks, Doc-UFCN was the best performer globally on this task, although far behind Mask-RCNN (AP values ranged from 0.16 to 0.85). This suggests that Doc-UFCN, as a semantic segmentation network, is the better competitor to Mask-RCNN, as an instance segmentation network. We will focus only on this network in the second study. Note also that both networks, Mask-RCNN and Doc-UFCN, showed their best performance on the HOME-Alcar database and their worst performance on the cBaD READ-complex dataset.

### 4.2. Study 2: Mask-RCNN against Doc-UFCN on the DRoSB Dataset

**Figure 9:**
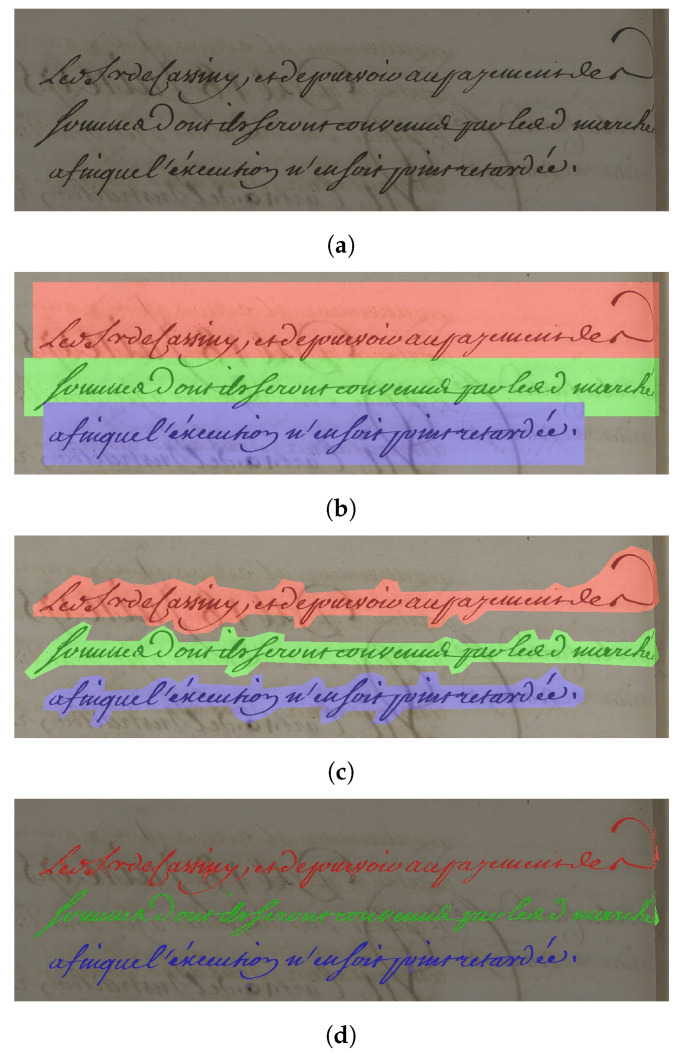
Different approaches of segmentation. (**a**) Example of text line; (**b**) bounding box approach; (**c**) mask approach; (**d**) pixels of line level obtained after light mask processing. (Applied to text lines from the DRoSB database, courtesy of the Archives of the Côte d’Or Departmental Council, Copyright 2023 (©/CD21/F.PETOT/2023)).

To better demonstrate the interest of using an instance segmentation network like Mask-RCNN, it is worth continuing the investigation with our private database, following the two approaches of the evaluation process (see Section 3.3.3), with and without the application of the LMP. cBaD 2017’s annotation style is governed by the use of bounding boxes. However, this seems to be irrelevant in the case of text line segmentation for text transcription. In fact, it may actually degrade performance. Useful pixels are those that belong to the lines and only those. The bounding boxes (Figure 9b) extracted from native images (Figure 9a) have arbitrary shapes that do not exactly match the line shape and may contain noisy data or, on the contrary, omit relevant pixels. In contrast, as shown in Figure 9c, the segmentation results obtained with a mask approach are more accurate. We therefore decided to use, in the second study, a new database that allows a mask approach, namely, the DRoSB dataset. To challenge Mask-RCNN on this database, Doc-UFCN was chosen because it was the network that achieved the best global performance on the large variety of databases tested by [17], and was the closest to Mask-RCNN in our first study when using object-level metrics. This semantic segmentation network was, therefore, the best challenger for an instance segmentation like Mask-RCNN.

In the following, we evaluate Mask-RCNN and Doc-UFCN, on the one hand, with their respective raw mask outputs (no LMP) and, on the other hand, after applying the LMP described in Section 3.3.3 in order to highlight the useful pixels while keeping out the spurious pixels, as shown in Figure 9d. In order to create the ground truths, we annotated 135 double-page spreads of the DRoSB dataset, each double page containing an average of 40 lines per page. Because this database had a limited number of pages, we performed a K-fold cross-validation with k = 5 to obtain an 80/20 split between training and evaluation. Table 3 presents the results for the pixel-level metrics.

Table 3 indicates that if we compare the raw outputs of Mask-RCNN and Doc-UFCN (no LMP) on our private database, Mask-RCNN obtains a higher IoU (0.71) and a slightly higher F1-score (0.82) than Doc-UFCN (0.65 and 0.78, respectively), showing that it fits the expected masks better. When we apply the LMP algorithm to the masks generated by each of these networks, Mask-RCNN performs clearly better (0.84 and 0.91, respectively) than Doc-UFCN (0.55 and 0.70, respectively). The decrease in Doc-UFCN’s performance with LMP shows that this network produces errors mainly in areas containing relevant pixels by including irrelevant pixels. In fact, the wider the masks (and they are necessarily wider with Doc-UFCN than with Mask-RCNN), the greater the risk of including pixels belonging to a line other than the one being segmented (the one above or below). On the contrary, the increase in performance of Mask-RCNN with LMP shows that the errors of this network are mainly produced in areas containing irrelevant pixels (empty areas).

Table 4 presents the results for the object-level metrics. It confirms that Mask-RCNN outperforms Doc-UFCN on every metric. Without LMP, the AP@0.5 is 0.90 for Mask-RCNN versus 0.61 for Doc-UFCN, the AP@0.75 is 0.46 versus 0.18, and, finally, the AP[0.5,0.95] is 0.48 versus 0.26. Applying LMP leads to a valuable increase in the AP values for both networks; however, it maintains the superiority of Mask-RCNN over Doc-UFCN for all AP measures. Furthermore, for Mask-RCNN, the standard deviations are largely reduced with LMP. The higher values of the standard deviations without LMP effectively attest to the variability of human annotations. The benefit of LMP appears to be even greater for Doc-UFCN, with a significant increase in average performance measures while maintaining low standard deviations.

### 4.3. Study 3: Mask-RCNN Segmentation against Doc-UFCN Segmentation on Transcription Performance

A real-world scenario context involves segmentation followed by text transcription. In fact, the ultimate goal of historical document analysis is handwritten text recognition (HTR). Therefore, we conducted an experiment on the complete processing chain: line segmentation followed by transcription of the current line using an HTR model. We chose TrOCR [36], because it is widely recognized as one of the best transcription models. This model is based on an encoder–decoder transformer. In the available version of the network, the encoder was pretrained on ImageNet [37] and the decoder on a masked language model. Then, the entire model was trained on the IAM dataset [38]. For our own use, we “fine-tuned” this model on the DRoSB database. To calculate the character error rate (CER), we reconstructed the segmented page using the positions of the masks in the image. We considered two masks to be part of the same line if they were at the same height in the image, within a margin of 30% of the size of a line.

The results showed a better performance for Mask-RCNN. In fact, the CER with the Mask-RCNN segmentation was 4.2%, while this error rate was 15.3% for Doc-UFCN segmentation. However, it is important to note that some of the errors associated with Doc-UFCN were due to small data-free zones. To remedy this, we eliminated areas below a certain size (45 square pixels), which reduced Doc-UFCN’s error rate from 15.3% to 13.1%. Mask-RCNN’s performance remained constant under these conditions. Thus, even if we try to improve the performance of Doc-UFCN, Mask-RCNN outperforms Doc-UFCN in terms of transcription performance. Note that this HTR performance of Doc-UFCN is close to the best ones reported by [17] with several databases. In fact, the two lowest CERs found by these authors for this network were 11.7% (Bozen [39] dataset) and 14.8% (Horae [40] dataset), showing that our results for Doc-UFCN are reasonable. None of the other U-Net networks tested by [17] had a CER lower than 11.7%. In our opinion, the clear superiority of Mask-RCNN in the HTR performance definitively confirms its high efficiency as a text line segmenter in the case of historical documents.

## 5. Discussion and Conclusions

The present paper is the first to directly compare Mask-RCNN (as an example of instance segmentation network) with state-of-the art U-Net networks (as examples of semantic segmentation networks) for line segmentation in historical documents. Mask-RCNN clearly outperforms U-Net networks in its ability to separate close but distinct lines, and, thus, in its HTR performance. U-Net-based models require too many adjustments, especially in post-processing, to be well suited to the line segmentation task for challenging documents such as historical ones. These results are in line with the observations reported in a study in the medical domain [25], in which Mask-RCNN appeared to be optimal for separating close objects, while U-Net was better at detecting objects but tended to merge close objects. Also in our first study, ARU-Net was more efficient than Mask-RCNN at detecting the pixels of the lines in almost all the databases tested. When a more suitable database for the mask approach developed in Mask-RCNN was used, as in the second study with the DRoSB database, Mask-RCNN confirmed its efficiency, attaining an AP[0.5,0.95] value of 0.78, which is rather satisfactory (and of course higher than that achieved by the best U-Net competitor network in our study conditions).

An interesting innovation proposed in the present work is the development of a simple algorithm (LMP) for generating ground truths, which reduces the risk of introducing bias during training and evaluation. LMP is applied to existing measures in the literature and ensures more accurate estimates of real network performance. In other words, LMP increases the precision with which measures are made. Therefore, it naturally benefits both Mask-RCNN and Doc-UFCN, as shown by the results of Study 2. In fact, these results validate the original solution proposed to avoid the use of raw masks for the evaluation, by subjecting them to light processing consisting of selecting only the relevant pixels. It should be noted that this LMP can be applied independently of the database used.

The ultimate demonstration of the efficiency of the Mask-RCNN approach compared to the U-Net approach instantiated by Doc-UFCN was brought about by Study 3. In this study, the output of both networks fed a neural network dedicated to transcription, precisely a Transformer network [41]. The results show better transcription performance for Mask-RCNN, with only 4% of CER, while the error rate for Doc-UFCN segmentation reaches 13% after optimization. Thus, Mask-RCNN offers competitive performance with reasonable computational requirements, demonstrating its practicality in real-world text segmentation and transcription scenarios. Indeed, the line segmentation algorithm developed here can be easily integrated into any type of collaborative platform like, for instance, eScriptorium (https://escriptorium.fr/ (accessed on 1 June 2023)). To facilitate its use in the context of the institution that owns the DRoSB dataset, we developed a specific platform that allows paleographers or hobbyists to directly segment scanned digital images of historical texts into lines and transcribe their content.

Of course, further research is needed to progress in this domain of text line segmentation of historical documents. On the one hand, Mask-RCNN can still be better optimized. For example, the Inception V2 backbone present in Mask-RCNN could be replaced with the ConvNeXt network [42], which would certainly improve the accuracy and processing speed of the network. On the other hand, it might be worthwhile to test a “proposal-free” instance segmentation network ([15]), such as SOLOv2 ([29]), on a large variety of historical documents in comparison to a “proposal-based” network such as Mask-RCNN. It is an empirical question to know which of the clustering/grouping or bounding box before segmentation approaches to mask generation would adapt better to very different handwriting families. Such an investigation could not be included in the present work, which had a different objective. It warrants a separate study, which we are currently planning.

## Figures and Tables

**Table 1:** Pixel-level metrics on cBaD 2017 READ-Complex, DIVA-HisDB, and HOME-Alcar (1: performance values drawn from [17]; 2: performance values drawn from [33]).

	IoU			F1-Score		
**Network**	**cBaD**	**DIVA**	**HOME**	**cBaD**	**DIVA**	**HOME**
ARU-Net ^1^	0.73	0.60	0.67	0.81	0.96	0.94
dhSegment ^1^	0.58	0.46	0.55	0.73	0.60	0.73
Doc-UFCN ^1^	0.49	0.67	0.60	0.70	0.80	0.77
Dilated-FCN ^2^	-	-	-	0.75	0.92	-
Mask-RCNN	0.64	0.55	0.85	0.76	0.70	0.91

**Table 2:** Object-level metrics on cBaD 2017 READ-Complex, DIVA-HisDB, and HOME-Alcar (1: performance values drawn from [17]).

	AP@0.5			AP@0.75			AP@[0.5,0.95]		
**Network**	**cBaD**	**DIVA**	**HOME**	**cBaD**	**DIVA**	**HOME**	**cBaD**	**DIVA**	**HOME**
ARU-Net ^1^	0.22	0.10	0.19	0.08	0.03	0.00	0.08	0.04	0.04
dhSegment ^1^	0.62	0.39	0.78	0.15	0.11	0.12	0.24	0.17	0.28
Doc-UFCN ^1^	0.61	0.77	0.85	0.16	0.33	0.49	0.24	0.36	0.46
Mask-RCNN	0.87	0.96	0.98	0.34	0.60	0.86	0.42	0.55	0.65

**Table 3:** IoU and F1-score (pixel-level metrics) of Doc-UFCN and Mask-RCNN networks for the DRoSB dataset with and without application of the light mask processing (LMP) operation. Standard deviations in brackets.

Network	LMP	R	P	IoU	F1-Score
Doc-UFCN	No	0.73 (0.04)	0.88 (0.06)	0.65 (0.03)	0.78 (0.03)
Mask-RCNN	No	0.83 (0.08)	0.85 (0.07)	0.71 (0.05)	0.82 (0.03)
Doc-UFCN	Yes	0.92 (0.04)	0.60 (0.06)	0.55 (0.06)	0.70 (0.05)
Mask-RCNN	Yes	0.89 (0.04)	0.94 (0.02)	0.84 (0.04)	0.91 (0.02)

**Table 4:** Average precision (object level metrics) of Doc-UFCN and Mask-RCNN on the DRoSB dataset with and without application of the light mask processing (LMP) operation. Standard deviations are in brackets.

Network	LMP	AP@0.5	AP@0.75	AP@[0.5,0.95]
Doc-UFCN	No	0.61 (0.06)	0.18 (0.08)	0.26 (0.04)
Mask-RCNN	No	0.90 (0.13)	0.46 (0.21)	0.48 (0.10)
Doc-UFCN	Yes	0.62 (0.08)	0.52 (0.08)	0.50 (0.08)
Mask-RCNN	Yes	0.96 (0.02)	0.85 (0.06)	0.78 (0.06)

## Data Availability

The DRoSD dataset used in this article has been made publicly available (https://zenodo.org/records/10725540 (accessed on 26 February 2024)).

## References

[1] Archives F.N. (1997). Gallica.

[2] Paillard J., Nadeau C., Haliwell W., Roberts K., Roberts G. (1980). Nouveaux objectifs pour l’étude de la performance motrice intégrée: Les niveaux de contrôle. Psychology of Motor Behavior and Sport.

[3] Likforman-Sulem L., Zahour A., Taconet B. (2007). Text line segmentation of historical documents: A survey. Int. J. Doc. Anal. Recognit. (IJDAR).

[4] Diem M., Kleber F., Fiel S., Gruning T., Gatos B. cBAD: ICDAR2017 Competition on Baseline Detection. Proceedings of the 2017 14th IAPR International Conference on Document Analysis and Recognition (ICDAR).

[5] Kurar Barakat B., Cohen R., Droby A., Rabaev I., El-Sana J. (2020). Learning-Free Text Line Segmentation for Historical Handwritten Documents. Appl. Sci..

[6] Nguyen T.N., Burie J.C., Le T.L., Schweyer A.V. An effective method for text line segmentation in historical document images. Proceedings of the 2022 26th International Conference on Pattern Recognition (ICPR).

[7] Lecun Y., Bottou L., Bengio Y., Haffner P. (1998). Gradient-Based Learning Applied to Document Recognition. Proc. IEEE.

[8] Minaee S., Boykov Y., Porikli F., Plaza A., Kehtarnavaz N., Terzopoulos D. (2022). Image Segmentation Using Deep Learning: A Survey. IEEE Trans. Pattern Anal. Mach. Intell..

[9] Redmon J., Divvala S., Girshick R., Farhadi A. (2016). You Only Look Once: Unified, Real-Time Object Detection. arXiv.

[10] Liu W., Anguelov D., Erhan D., Szegedy C., Reed S., Fu C.Y., Berg A.C. (2016). SSD: Single Shot MultiBox Detector. arXiv.

[11] Girshick R. (2015). Fast R-CNN. arXiv.

[12] Clérice T. (2022). You Actually Look Twice At it (YALTAi): Using an object detection approach instead of region segmentation within the Kraken engine. arXiv.

[13] Ronneberger O., Fischer P., Brox T. (2015). U-Net: Convolutional Networks for Biomedical Image Segmentation. arXiv.

[14] He K., Gkioxari G., Dollár P., Girshick R. (2018). Mask R-CNN. arXiv.

[15] Sharma R., Saqib M., Lin C.T., Blumenstein M. (2022). A Survey on Object Instance Segmentation. SN Comput. Sci..

[16] Droby A., Kurar Barakat B., Alaasam R., Madi B., Rabaev I., El-Sana J. (2022). Text Line Extraction in Historical Documents Using Mask R-CNN. Signals.

[17] Boillet M., Kermorvant C., Paquet T. (2022). Robust text line detection in historical documents: Learning and evaluation methods. Int. J. Doc. Anal. Recognit. (IJDAR).

[18] Simistira F., Seuret M., Eichenberger N., Garz A., Liwicki M., Ingold R. DIVA-HisDB: A Precisely Annotated Large Dataset of Challenging Medieval Manuscripts. Proceedings of the 2016 15th International Conference on Frontiers in Handwriting Recognition (ICFHR).

[19] Stutzmann D., Torres Aguilar S., Chaffenet P. (2021). HOME-Alcar: Aligned and Annotated Cartularies.

[20] Oliveira S.A., Seguin B., Kaplan F. dhSegment: A generic deep-learning approach for document segmentation. Proceedings of the 2018 16th International Conference on Frontiers in Handwriting Recognition (ICFHR).

[21] Grüning T., Leifert G., Strauß T., Michael J., Labahn R. (2019). A Two-Stage Method for Text Line Detection in Historical Documents. Int. J. Doc. Anal. Recognit. (IJDAR).

[22] Boillet M., Maarand M., Paquet T., Kermorvant C. Including Keyword Position in Image-based Models for Act Segmentation of Historical Registers. Proceedings of the 6th International Workshop on Historical Document Imaging and Processing.

[23] Renton G., Chatelain C., Adam S., Kermorvant C., Paquet T. Handwritten Text Line Segmentation Using Fully Convolutional Network. Proceedings of the 2017 14th IAPR International Conference on Document Analysis and Recognition (ICDAR).

[24] Ren S., He K., Girshick R., Sun J. (2016). Faster R-CNN: Towards Real-Time Object Detection with Region Proposal Networks. arXiv.

[25] Vuola A.O., Akram S.U., Kannala J. (2019). Mask-RCNN and U-net Ensembled for Nuclei Segmentation. arXiv.

[26] Marechal E., Jaugey A., Tarris G., Martin L., Paindavoine M., Rebibou J., Mathieu L. (2022). FC046: Automated Mest-C Classification in IGA Nephropathy using Deep-Learning based Segmentation. Nephrol. Dial. Transplant..

[27] Van Wymelbeke-Delannoy V., Juhel C., Bole H., Sow A.K., Guyot C., Belbaghdadi F., Brousse O., Paindavoine M. (2022). A Cross-Sectional Reproducibility Study of a Standard Camera Sensor Using Artificial Intelligence to Assess Food Items: The FoodIntech Project. Nutrients.

[28] Zhao P., Wang W., Cai Z., Zhang G., Lu Y. (2021). Accurate Fine-grained Layout Analysis for the Historical Tibetan Document Based on the Instance Segmentation. IEEE Access.

[29] Wang X., Zhang R., Kong T., Li L., Shen C. (2020). SOLOv2: Dynamic and Fast Instance Segmentation. arXiv.

[30] Fizaine F.C., Robin C., Paindavoine M. Transcription Automatique de textes du XVIIIe siècle à l’aide de l’intelligence artificielle. Proceedings of the Conference of AI4LAM Les Futurs Fantastiques.

[31] Fizaine F.C., Bouyé E. Lettres en Lumières. Proceedings of the Conference of CremmaLab Documents Anciens et Reconnaissance Automatique des éCritures Manuscrites.

[32] Ostu N. (1979). A Threshold Selection Method from Gray-Level Histograms. IEEE Trans. Syst. Man, Cybern..

[33] Mechi O., Mehri M., Ingold R., Essoukri Ben Amara N. Text Line Segmentation in Historical Document Images Using an Adaptive U-Net Architecture. Proceedings of the 2019 International Conference on Document Analysis and Recognition (ICDAR).

[34] Everingham M., Van Gool L., Williams C.K.I., Winn J., Zisserman A. (2010). The Pascal Visual Object Classes (VOC) Challenge. Int. J. Comput. Vis..

[35] Wick C., Puppe F. Fully Convolutional Neural Networks for Page Segmentation of Historical Document Images. Proceedings of the 2018 13th IAPR International Workshop on Document Analysis Systems (DAS).

[36] Li M., Lv T., Cui L., Lu Y., Florencio D., Zhang C., Li Z., Wei F. (2021). TrOCR: Transformer-based Optical Character Recognition with Pre-trained Models. arXiv.

[37] Deng J., Dong W., Socher R., Li L.J., Li K., Fei-Fei L. ImageNet: A large-scale hierarchical image database. Proceedings of the 2009 IEEE Conference on Computer Vision and Pattern Recognition.

[38] Marti U.V., Bunke H. (2002). The IAM-database: An English sentence database for offline handwriting recognition. Int. J. Doc. Anal. Recognit..

[39] Sánchez J.A., Romero V., Toselli A.H., Vidal E. (2016). Bozen Dataset.

[40] Boillet M., Bonhomme M.L., Stutzmann D., Kermorvant C. HORAE: An annotated dataset of books of hours. Proceedings of the 5th International Workshop on Historical Document Imaging and Processing.

[41] Vaswani A., Shazeer N., Parmar N., Uszkoreit J., Jones L., Gomez A.N., Kaiser L., Polosukhin I. (2017). Attention Is All You Need. arXiv.

[42] Liu Z., Mao H., Wu C.Y., Feichtenhofer C., Darrell T., Xie S. (2022). A ConvNet for the 2020s. arXiv.

